# Identifying ways to maximise cervical screening uptake: a qualitative study of GPs’ and practice nurses’ cervical cancer screening-related behaviours

**DOI:** 10.12688/hrbopenres.13246.1

**Published:** 2021-05-05

**Authors:** Mairead O'Connor, Lisa A. McSherry, Stephan U. Dombrowski, Jill J. Francis, Cara M. Martin, John J. O'Leary, Linda Sharp

**Affiliations:** 1 School of Public Health, Univesity College Cork, Cork, Ireland; 2 National Cancer Registry of Ireland, Kinsale Road, Cork, Ireland, Ireland; 3 Faculty of Kinesiology, University of New Brunswick, Fredericton, NB, Canada; 4 School of Health Sciences, City University London, London,, UK; 5 Trinity College Dublin, Dublin, Ireland; 6 Institute of Health and Society, Newcastle University, Newcastle, UK

**Keywords:** cervical screening, women, primary care practitioners, clinical behaviours, the Theoretical Domains Framework

## Abstract

**Background:  **Cervical screening uptake is declining in several countries. Primary care practitioners could play a greater role in maximising uptake, but better understanding is needed of practitioners’ cervical screening-related behaviours. Among general practitioners (GPs) and practice nurses, we aimed to identify cervical screening-related clinical behaviours; clarify practitioners’ roles/responsibilities; and determine factors likely to influence clinical behaviours.

**Methods: **Telephone interviews were conducted with GPs and practice nurses in Ireland. Interview transcripts were analysed using the Theoretical Domains Framework (TDF), a comprehensive psychological framework of factors influencing clinical behaviour.

**Results: **14 GPs and 19 practice nurses participated. Key clinical behaviours identified were offering smears and encouraging women to attend for smears. Smeartaking responsibility was considered a predominantly female role. Of 12 possible theoretical domains, 11 were identified in relation to these behaviours. Those judged to be the most important were beliefs about capabilities; environmental context and resources; social influences; and behavioural regulation. Difficulties in obtaining smears from certain subgroups of women and inexperience of some GPs in smeartaking arose in relation to beliefs about capabilities. The need for public health education and reluctance of male practitioners to discuss cervical screening with female patients emerged in relation to social influences.

**Conclusions:** We identified - for the first time - primary care practitioners’ cervical-screening related clinical behaviours, their perceived roles and responsibilities, and factors likely to influence behaviours. The results could inform initiatives to enable practitioners to encourage women to have smear tests which in turn, may help increase cervical screening uptake.

## Introduction

Well-organised cervical screening is effective in reducing cervical cancer incidence and mortality ^1, 2^. One of the keys to screening success is maximising uptake ^3^. Thus, it is concerning that screening uptake is declining in several developed countries, including England, Sweden, and Australia ^4^. For example, in England, the proportion of eligible women being screened has declined from 76% in 2011 to 72% in 2017 ^5^. In the U.S, a downward trend in smear test use has been shown from 2000 to 2015 ^6^. There is currently considerable interest in developing initiatives and strategies to reverse these concerning trends. For example, the Cervical Screening Programme (CSP) in England has affirmed that halting this trend is a key programme objective and are supporting research in this area ^7^.

Cervical screening is a significant component of the primary care workload. Evidence is emerging that the screening-related attitudes and clinical behaviours of primary care practitioners (e.g. general practitioners (GPs), practice nurses) influence women’s cervical screening behaviours. For example, a previous bad experience in attending for a smear deters women from attending for another smear ^8, 9^. In contrast, a considerate smear taker who takes care to minimise pain and discomfort may positively influence women’s future participation. Moreover, women report that GP prompting, and a positive GP attitude, are important motivators for having smears ^10^, while a negative or dismissive GP attitude is a significant barrier ^11^, findings replicated in various settings ^12, 13^.

To date, the evidence of practitioners’ influences on screening uptake has concentrated on doctors. Practice nurses are increasingly involved in delivering cervical screening in several settings, including the UK, Ireland and Australia ^14– 16^. It seems likely, therefore, that practice nurses may influence women’s screening-related behaviours in similar ways to GPs ^17– 19^.

The influence of primary care practitioners on women’s behaviours raises the possibility that there may be opportunities to engage practitioners more actively and/or directly in maximising cervical screening uptake. As a first step, there is a need to better understand what practitioners consider to be their responsibilities with regard to screening and what factors may influence their screening-related clinical behaviours. We undertook a qualitative study with general practitioners (GPs) and practice nurses to explore different clinical behaviours around cervical cancer prevention - HPV testing, HPV vaccination and smear tests/cytological screening. The results on HPV-related clinical behaviours (e.g. initiating a discussion about HPV with female patients, recommending HPV vaccination) have been previously published ^20^. The current paper focuses on results related to cervical screening related clinical behaviours with the following aims: to: (1) identify cervical screening-related clinical behaviours; (2) clarify practitioners’ roles and what they consider to be their responsibilities in relation to cervical screening; and (3) determine factors likely to influence their cervical-screening related clinical behaviours.

## Methods

### Setting

The study setting was Ireland which has a mixed public/private healthcare system. At the time the study was conducted, Ireland’s health service (the Health Service Executive (HSE)) was divided up into four geographical health board areas covering the Republic of Ireland (HSE Mid-Eastern, HSE North-Eastern, HSE Southern and HSE Western). GPs are effectively private practitioners but may provide services for certain patients (typically those with low incomes) under the public system for which they are reimbursed by the state ^21^. The national screening programme, CervicalCheck, began in 2008 and offers free cervical screening for women aged 25–65 years in primary care. Women are invited, through a centralised call-recall system. A woman may choose to have a screening test (at her general practice or with any other registered screening test taker). CervicalCheck reimburses practitioners for screening tests taken within guidelines; additional/opportunistic screening tests are not reimbursed. There are currently no screening uptake targets for individual practices, but nationally, the programme achieved 75% coverage of the eligible population in its first five years of operation ^22^. Until very recently, the programme used smear tests as its primary screening tool – women were invited for smear tests every 3 years (for women aged 25–44) or 5 years (for older women). Traditionally, GPs were the primary smeartakers, but now, practice nurses carry out the vast majority of cervical screening tests within CervicalCheck ^23^, although GPs retain clinical responsibility for the provision of the service in their practice. In March 2020, the programme began replacing traditional cervical cytology (smear) with primary HPV testing in line with best international recommendations – now women aged 25–29 years are screened every 3 years and those aged 30–65 are screened every 5 years. Irrespective of the recent changes to the screening programme, the overall screening process from the point of view of both the woman and the test taker (e.g. registering as a screening test taker, a woman choosing where to have her screening test) remains largely unchanged.

### Participants

GPs and practice nurses working in Ireland were eligible to participate. GPs were recruited via postal invitation from a group of 145 GPs who had: been randomly sampled from a national GP database (comprising approximately 2,000 registered GPs in Ireland), participated in a cervical screening survey in 2007, and indicated they were potentially willing to assist with further research ^24^. The group was diverse in terms of personal and practice characteristics (in 2007). A purposive sample was recruited from this group of 145 GPs. Sampling strata was defined in terms of variables that had been found in the 2007 survey to be strongly associated with attitudes towards smear taking. These variables were: GP gender, years since graduation, area of practice location (HSE health board area). Since there is no national practice nurse database two routes were used to recruit nurses to ensure maximum sample variation. Attendees at a national Irish Practice Nurses Association (IPNA) conference were invited to participate and postal invitations were dispatched to randomly selected nurses via area-based practice nurse professional development coordinators (PDCs) across Ireland. All practitioners who returned a reply slip were contacted for interview. Ethical approval was obtained from the Irish College of General Practitioners.

### Procedures

Semi-structured interviews took place between November 2010 and February 2011. Participants received an information sheet about the research and provided written informed consent before the interview commenced. Telephone interviews (lasting 16 to 50 minutes in duration) were conducted (by LAMcS, ATHENS research investigator. ATHENS - an intervention trial of HPV education and support in primary care) and guided by a topic guide (see extended data ^25^). The guide included open “core” questions and clinical scenarios designed to elicit information about cervical cancer screening-related clinical behaviours, roles/responsibilities, and drivers of clinical behaviours. The same core questions were asked of both GPs and practice nurses. The order in which the core questions were asked, and the content and order of follow-up questions and prompts, varied between practitioners.

Recruitment continued until new issues ceased to emerge for GPs and practice nurses separately (i.e. data saturation was reached in each practitioner group (practitioner group – (1) GPs or (2) practice nurses ^26^). All except three interviews were audio-recorded and transcribed verbatim (the three interviews were not audio-recorded as study participants declined consent for their interviews to be recorded). When interviews were not recorded, the interviewer took detailed notes contemporaneously. The study adhered to the Standards for reporting qualitative research: a synthesis of recommendations (SRQR) ^27^ (see extended data for completed SRQR checklist ^25^).

### Analysis

GP and practice nurse interviews were analysed collectively. No qualitative data software was used for analysis. Analysis was conducted following the Framework Analysis approach ^28, 29^, to identify key cervical screening-related clinical behaviours and roles/responsibilities of practitioners. We used the Theoretical Domains Framework (TDF) as the coding framework ^30^ to determine the factors likely to influence the behaviours. Developed through an expert consensus process, the TDF summarises multiple psychological and organisational theories regarding influences on clinical behaviour in 12 theoretical domains: *knowledge; skill, social/professional role and identity; beliefs about capabilities; beliefs about consequences; motivation and goals; memory, attention and decision processes; environmental context and resources; social influences; emotion; behavioural regulation;* and *nature of the behaviour.* It is a leading theoretical framework for developing interventions aimed at changing clinical behaviour. Two investigators (LAMcS, LS) read and reread all transcripts, independently coded these, combined codes into subthemes and allocated these, and participants’ direct quotes, to the TDF domains. For analytical rigour, a second iteration of this process was performed with uncertainties resolved in discussion with co-investigators with expertise in behaviour change (JJF, SUD). The domains (themes) considered strongest/dominant were those: (1) mentioned by most practitioners; (2) where most subthemes were identified; and (3) which were discussed at greatest length ^31^. Whether subthemes arose solely among male/female GPs, practice nurses or both was noted. Illustrative quotes are provided to supplement narrative descriptions. Study participants were not invited to provide feedback on the interview data but were sent a final report on study findings.

## Results

Of the 145 GPs contacted, 19 responded (i.e. returned a reply slip). All 19 GPs who responded were interviewed. Of the 30 practice nurses approached through PDCs, ten were interviewed; four nurses were recruited from the annual conference. Table 1 summarises participants’ characteristics.

**Table 1.** Characteristics of practitioners interviewed.

Characteristic		*GPs*	*Practice Nurses*
Sex	Female	*13*	*14*
	Male	6	0
Health board area	HSE Mid-Eastern	4	3
	HSE North-Eastern	3	4
	HSE Southern	7	0
	HSE Western	5	7
Location of practice	City	6	6
	Other	13	8
Solo GP practice	Yes	6	7
	No	13	7
Practice nurse(s) employed in practice	Yes	16	-
	No	3	-
Years since graduation ^1^	<10 years	2	-
	10–19 years	2	-
	20+ years	14	-

^1^from responses to 2007 GP survey

### Clinical behaviours

Two cervical screening-related clinical behaviours were identified among both GPs and practice nurses: (1) offering smears to women and (2) encouraging women to attend for smears.

### Roles and responsibilities

Taking smears was considered a predominantly female role with responsibility falling to female GPs and practice nurses (all of whom are female). Female practitioners frequently assumed that women prefer to have the option of a female smeartaker, making comments like “I do.....99% of them because the other two GPs are male (PN5020)” and “it’s a male doctor and a lot of the patients don’t seem to......[want to]....avail of a male (PN5040).” Male GPs performed smears less often and made comments like ”I do an occasional one when a patient requests it (GP0133).”

### Factors influencing Clinical behaviours

Table 2 displays the 12 theoretical domains, subthemes identified which relate to the individual domains, and illustrative quotes. All but one of the 12 theoretical domains, *motivation and goals,* influenced both offering smears and encouraging women to attend ( Table 2). The dominant domains were: *beliefs about capabilities*, *environmental context and resources*, *behavioural regulation* and *social influences*. These are described in more detail below.

**Table 2.** Factors influencing behaviours related to cervical screening.

Theme / construct domain	Subtheme / specific belief	Practitioner *		Sample quotes
		GPs	Practice Nurses	
**1. Knowledge**	Unaware that they can still take a smear if the patient is not already registered	ü	**-**	*'It also depends on whether she is actually registered with CervicalCheck, because we are being told they* *won't process smears unless they are registered' - GP 0058*
	Unaware that they can post 1–2 smear samples back at a time	ü	*-*	*''The cardboard boxes which are packed in sixes or fours I can't remember so there's kind of a posting issue* *that you kind of accumulate… get a few together for the post…. A small issue'' - GP 0133*
	Unaware that they can take a smear between days 10–20 of woman's cycle	**-**	ü	*"Sometimes the women arrive and they're not in the required time, they're not between day 10 and 20"* *- Practice nurse 5017*
**2. Skill**	Difficulty taking a smear from some patients e.g., obese, learning difficulties	ü	ü	*"Sometimes there are technical issues e.g. if you have a very obese patient or a patient with an arthritic hip* *that can be physically challenging" - GP 0136*
	Difficulty visualising the cervix	ü	ü	*"Occasionally it can be difficult to ur visualise the cervix" - GP 0090*
	Difficulty assessing whether a patient has been sexually active	**-**	ü	*"It's very hard sometimes to I suppose in one sense assess whether this person had been sexually active* *or not….. It sometimes can be a sensitive issue on bringing up whether they've been sexually active or not"* *- Practice nurse 5034*
	Ability to put patients at ease	ü	**-**	
	Ability to explain to patient what smear test is for & differentiate it from STI testing	ü	**-**	*''Often people come for smears and what they're really looking for is STI testing'' - GP 0003*
	GPs becoming deskilled	ü	**-**	*"I wouldn't be particularly upskilled at the moment" - GP 0026*
**3. Social /** **professional role** **& identity**	Role of smeartaker is shifting:			
	Male GPs less likely to take smears	ü	ü	*"Harder for male GPs as most women want to go to a female doctor" - GP 0016*
	GP role more auxillary i.e., nurse may refer to GP if complications arise	ü	ü	*''The nurse does the smears ur and we supervise'' - GP 0026*
	Feel responsible for recall	ü	**-**	*"It looks like it's down to us to make sure they're recalled for their second smear" - GP 0141*
	CervicalCheck training not mandatory for GPs	**-**	ü	
**4. Beliefs about** **capabilities**	Difficulty dealing with awkward or sensitive situations e.g. anxious woman	ü	ü	*"What can make it difficult? Well I guess mmm a difficult exam, just ur either patient anxiety or fretfulness"* *-GP 0026*
	Need for good local contacts for advice & to refer technically challenging smears	ü	ü	
	Difficulty getting women to attend for smears	ü	ü	*"The only problem is getting them to come in" - GP 0034*
	Difficulty dealing with women who have never been sexually active	ü	**-**	*"The only time I'd have difficulty is with is with somebody if they haven't been sexually active. Now it's not a* *barrier really I suppose but you have to tease that out obviously with the person" -GP 0072*
	GP reluctance to admit inexperience	**-**	ü	*''We've had people who've never taken a smear test before and you mightn't realise it. And sometimes* *people won't tell you that mmmm I think sometimes medical people are not as quick to say I've never done that before or I'm not 100% sure'' - Practice nurse 5001*
	Practitioners are no longer in control of the recall system	**-**	ü	*"What I found hard to come to terms with at the start was I felt I had control of the actual system" - Practice* * nurse 5040*
**5. Beliefs about** **consequences**	CervicalCheck not aware of patient history - caused some problems	ü	ü	*''We're told under 25 is not of much significance…. one would wonder about that with you know promiscuity* *at the rate it's at, at the moment with these young girls…'' - GP 0129*
	Belief that it is an unpleasant / intrusive test for women	ü	ü	*"I suppose the ideal thing would that mmm it wouldn't be a test you know the test itself is physically mmm* *unpleasant…. Ideally if you could swab from the mouth…. Or take a urine sample or something like that.* *But the actual having to get the sample from the place you have to get it is going to make it difficult" - GP* *0058*
	Belief that women who don't have a smear 'there and then' will not come back at another time	ü	ü	
	Belief that payment for taking smears is too low	ü	**-**	*"The payment (for smear taking) is derisory, it's ridiculous, I mean the amount of work and time that goes* *into it" - GP 0058*
**6. Motivation ** **and goals**	-	-	-	-
**7. Memory,** **attention** **and decision** **processes**	Having a reminder system for follow-ups	ü	ü	*''I actually keep the manual register as well so ur it means that if any smear result doesn’t come back I can* *see it'' - Practice nurse 5002*
**8. Environmental** **context and** **resources**	Don't have enough time to take smears	ü	ü	*"And I suppose time….. It's not difficult it's just time really" - GP 0058*
	Inadequate facilities / equipment	ü	ü	
	Limited practice staff support	ü	ü	*"I think it's having nursing time really. I think having more support" - GP 0060*
	Limited appointment times	ü	ü	*"Maybe times are a problem - their surgery is 9-5 so it might be difficult for women to get there if they are* *working" - Practice nurse 5026*
	Transport / accessibility issues for some patients	ü	ü	*"Transport here is a big thing for some people….. They live quite rural and they don't have a lift in at the* *last minute" - Practice nurse 5040*
	Lack of foreign language leaflets / language barriers	**-**	ü	*"Sometimes explaining to immigrant population is difficult" - Practice nurse 5036*
	Need better advertising of service	**-**	ü	
	Difficulty getting records for women with history of hysterectomy	**-**	ü	*'The big problem we're having a lot now is em women with histories of hysterectomies……….but the problem* *is getting back to the hospitals. Like some of these might have had their hysterectomy like 20 - 20 odd years* *ago. Em. And we don't know the full extent'' - Practice nurse 5004*
**9. Social influences**	Women not used to CervicalCheck programme e.g. recall system	ü	ü	*"Some women find it difficult to accept the recall system that's now in place" - GP 0129*
	Need for more public education	ü	ü	*"I think the general public need to be made more aware of this service" - GP 0129*
	Women don't know a lot about smears	ü	ü	*"I'm surprised at people's lack of knowledge" - GP 0003*
	Women choose who to go to for smears	ü	**-**	*"Patients just make an appointment for a smear with the doctor or nurse of their choice" -GP 0051*
	Women not used to nurse led service	ü	**-**	*"A lot of people aren't that use to the whole nurse thing you know" - GP 0060*
	Male GPs reluctant to broach topic with female patients	ü	**-**	*''I think generally speaking ur there is a hole there where single-handed GPs may may not broach the* *subject with female patients and the female patients may not feel comfortable going to the men''- GP 0072*
	Some women don't want to have smears taken then and there	**-**	ü	
	Women are not keen / afraid to come in for smears	**-**	ü	*"A lot of patients are actually afraid to come for a smear" - Practice nurse 5023*
**10. Emotion**	Frustration because of difficulty in getting smear from some patients	**-**	ü	*"There’s nothing more frustrating you have, twenty minutes to do a smear and you can’t find the cervix"* *- Practice nurse 5023*
	Awkwardness of practitioner (if woman outside of programme age range wants a smear)	ü	**-**	*"It's just awkward" -GP 0003*
**11. Behavioural** **regulation**	Belief that patients under 25 years should have access to the programme	ü	ü	
	Belief that not allowing opportunistic smeartaking was a mistake	ü	ü	*"I do think there is a bit more of a place for somebody just doing mmm and opportunistic smear. That we're* *no longer able to do" - GP 0046*
	Concern about recall period	ü	ü	
	Offering appointment system / dedicated smear clinics	ü	ü	*''I facilitate working ladies maybe on Saturday evening if I'm working duty for the weekend… we do* *everything to facilitate the appointment" - GP 0129*
	Having a reminder system for follow- ups	ü	ü	*"Send her a text to remind her to go (for follow-up test}" - Practice nurse 5017*
	Offering extended clinic opening hours	ü	-	
	Having leaflets available to give to patients	ü	ü	*"I give the ladies a leaflet explaining the results before when they come and do their smear" - Practice nurse* *5034*
	Having posters and signs advertising the service	-	ü	*"We have posters up in the, still up in the clinic" - GP 0060*
	Having "Flipcharts" to aid patient consultations	-	ü	*"It's a cardboard chart with like a ring thing at the top and it just explains what a smear test is…but it's quite* *simplified in fairness for the patients" - Practice nurse 5003*
	Having good secretarial staff to book clinics and liaise with women	ü	-	
	Having clearer CervicalCheck guidelines	ü	-	*"If there were much clearer guidelines…. What was set out as protocol and has actually come in are quite* *different" - GP 0141*
	Having a standard set of letters to cover every eventuality	ü	-	
	Having access to records for women with a history of hysterectomy	-	ü	
	More consistency in what staff record on smear forms (can affect follow-up recommendations)	ü	-	*Some people mightn't put down as much information on the form as others say in the surgery and it could* *come back like a three year recall for by rights when it should have been on an annual recall" - GP 0141*
	Team meetings involving nurses and GPs	ü	-	
**12. Nature of the** **behaviour**	Take a lot of smears	ü	-	
	Girls starting sexual behaviour at an earlier age	-	ü	*"In my, my bigger surgery mmm the, the women, well the girls, they start having sex a lot younger" -* *Practice nurse 5017*

* ü= mentioned by at least one practitioner

- = not mentioned by any practitioners

In terms of *beliefs about capabilities*, both practitioner groups spoke about difficulties in getting women to attend for smears. Once patients attend, practitioners reported difficulties in dealing with certain subgroups ( *e.g.,* women who are extremely anxious or have a history of sexual abuse). This was mentioned particularly frequently by female GPs. Some practice nurses described referring any cases expected to be “difficult” to GPs. One practice nurse noted that GPs may be reluctant to admit inexperience in smeartaking, which may have implications for quality assurance.

A lack of time to take smears, inadequate facilities and equipment, limited appointment times and limited practice support staff emerged in relation to *environmental context and resources*. Practitioners also noted that some women have accessibility issues making it difficult for them to attend the practice. Some practice nurses mentioned that difficulties arise because CervicalCheck does not hold women’s full smear and gynaecological histories (the programme databases only hold information from 2008 onwards). For example, CervicalCheck may be unaware that a woman has had a hysterectomy and may send her a smear invitation. GPs expressed a desire for clearer CervicalCheck guidelines on patient follow up after an abnormal smear.

In terms of behavioural regulation, some practitioners recognised opportunities to increase uptake by offering dedicated smear clinics and extended practice hours. Practice nurses suggested practical tools, such as flipcharts to use during consultations or clinic posters advertising the service, as potential means of improving the service. Many practitioners wanted to have the option of offering free smears to women outside the screening age range, if the practitioner felt this was warranted ( *e.g.,* for some women under 25). Furthermore, some criticised the policy of discouraging opportunistic smears at the practitioners’ discretion, and expressed concern that the recommended recall period was too long.

As regards *social influences*, both GPs and practice nurses believed there is a need for more public education around smears; they considered that many women do not know much about screening or smear tests, in part because the programme is relatively new. Practice nurses recognised that some women are not keen, or may be afraid, to attend for smears. GPs considered that male GPs may be reluctant to broach the topic of smears with female patients. They also noted that women are not yet familiar with nurse-led services and this may discourage women from attending.

## Discussion

### Summary of main findings

The major cervical screening-related clinical behaviours identified among primary care practitioners were offering smears and encouraging women to attend for smears. Responsibility for taking smears was considered a predominantly female role, with male GPs taking less responsibility for this task. Of the 12 theoretical domains, 11 were judged to influence clinical practice. This perhaps indicates that practitioners consider cervical screening to be a complex issue, influenced by multiple factors. The domains judged to be the most important behavioural influences were *beliefs about capabilities*, *environmental context and resources*, *social influences* and *behavioural regulation*.

### Comparison with existing literature

It was possible that practitioners, especially practice nurses, might have seen their role in relation to cervical screening as being limited to providing a service ( *i.e*., taking smears) for CervicalCheck. In fact, nurses and female GPs considered that encouraging women to attend for smears also formed part of their role. This suggests that female practitioners may be willing to engage with initiatives to enhance screening uptake. It might also have been expected *a priori* that practitioners would consider providing patients with information about screening or smears to be part of their role, but this did not emerge from the interviews. It would be interesting to see if this also applies in other settings. 

The move by male GPs away from responsibility for smeartaking – which emerged in several interviews with both GPs and practice nurses - was striking. In addition, it was particularly difficult to get male GPs to participate in the study, in part because (as they told us when they declined to take part) they viewed smeartaking as outside their remit. Others have shown that women have strong preferences for female smeartakers and are more likely to attend for screening with female GPs and practice nurses ^32, 33^. This is consistent with the assumptions of practitioners in this study that women prefer a female smeartaker. As regards maximising screening uptake, it is important that women receive consistent messages and encouragement from GPs; this implies that male GPs should be involved in strategies aimed at encouraging women to have smears, even if they do not take smears themselves. However, the findings of this study suggest that engaging male GPs with such strategies may prove difficult. 

Previous research has shown that women with a history of sexual abuse are less likely to attend for cervical screening ^34^. Our findings indicate that smear consultations with such women (and other “challenging” groups, such as women with learning difficulties) are also perceived as difficult by practitioners. While this may be unique to Ireland because of the relative infancy of CervicalCheck, it is also possible that the increasingly dominant role of practice nurses in smeartaking means that GPs’ belief in their own capabilities in these more challenging situations is declining. Since women who have a bad experience when having a smear may not reengage with the service ^8, 9^, it is important to ensure that practitioners have the skills and confidence to manage these types of consultations; support, training and/or interventions for practitioners in this area are, therefore, warranted.

Practical issues, such as limited appointment times and inadequate facilities, impacted on practitioners’ clinical behaviours. These, and similar issues, have also emerged as reasons why women fail to attend for smear tests or follow-up of abnormal smear results. For example, women cite difficulties in getting an appointment that fits with work/childcare commitments as a barrier to cervical screening attendance ^9, 33^. Providing convenient appointment times – perhaps outwith usual hours – may be a simple way in which screening uptake could be enhanced. 

In terms of the screening protocol in Ireland, there is little evidence that screening women under 25 years is effective ^35^. In our study, practitioners seemed to understand this but some– GPs especially –felt that they should have discretion to take a smear in a younger woman if they considered it warranted. In the UK the degree to which practitioners encourage women to attend for smears is related to their attitudes to the national cervical screening programme ^36^. In Ireland it remains to be seen whether practitioners who do not fully agree with CervicalCheck guidelines will actively encourage women to have smears and/or comply with strategies to enhance uptake. 

For women, emotional issues (such as embarrassment) impact on screening participation ^9^, but these did not arise as a major issue for most practitioners. This is unsurprising since practitioners have been trained not to talk about sexual behaviour in relation to smears, which probably removes much of the potential for embarrassment/awkwardness from the consultation. However, the increasing integration of HPV testing into screening may present difficulties. Some practitioners are uncomfortable discussing the sexual behaviour aspects of HPV in relation to cervical screening ^24^ and this may hinder future strategies designed to maximise uptake.

Around the time CervicalCheck was established, in 2008, there was some controversy over the level of reimbursement that would be provided by the programme to practitioners for taking smears: it was perceived as too low by some GPs who made their views well known. In light of this, it is noteworthy that payments did not emerge as an issue in relation to the domain of *motivation and goals*. This may reflect the fact that the level of reimbursement is now a *fait accompli* and the economic situation has changed dramatically. Alternatively, it is possible that any GPs who remain disgruntled with the payment simply declined to participate in this study. No other aspects of *motivation and goals* emerged as important influences on practitioners’ screening-related clinical behaviours. The most probable explanation for this is that, in contrast to the time before CervicalCheck, when smears were mainly taken opportunistically, a standard national screening protocol is now in place and practitioners are not responsible for call/recall. 

### Implications for practice

Most empirical research around increasing cervical screening uptake has focused on understanding women’s screening-related views. This study suggests that practitioners may be receptive to playing a more active role in encouraging women to have smears, and our findings in relation to the influences on practitioners’ behaviours could inform development of strategies or interventions to enhance these behaviours and, in turn, positively impact on uptake. Ensuring practitioners are comfortable communicating with patients about smears and that they have skills and capabilities to manage “difficult” smear related consultations could also help enhance uptake. Education and training in this area should target all practitioners, so that women who have a male GP are not disadvantaged. Cervical screening programmes are changing and are increasingly based on screening using HPV testing rather than cytology. The overall screening process will remain similar, despite the switch to HPV screening. For example, from the perspective of the woman undergoing an HPV test will be physically the same as having a smear test. However, GPs and practice nurses consider HPV a complex and challenging topic ^24^. In addition women are attached to and have confidence in smears, and concerns about programmes changing to HPV testing ^32, 37^. Practitioners need to be adequately supported and prepared for these changes, and the associated patient communication issues, so that screening uptake is not negatively affected.

### Strengths and limitations

The qualitative design allowed us to gain an in-depth understanding of the issues around cervical screening from practitioners’ perspectives. Data of such richness and depth would not have been available using a quantitative approach. The data may somewhat overemphasise experiences of practitioners with an interest in women’s health and views of practitioners with more positive attitudes towards screening than others since we interviewed GPs who had previously expressed an interest in this area. In addition, almost three quarters of the GPs interviewed had been in general practice for more than 20 years, but it is entirely unknown how this may influence their clinical practice and beliefs in relation to cervical screening. Overall, the interviewees’ characteristics were diverse and the process’ credibility and findings are evidenced in the diversity of themes and opinions which emerged. Despite practice nurses having responsibility for smeartaking in several healthcare systems, as far as we are aware, this is the first study to directly explore their roles and behaviours. Interviews for the study were conducted in late 2010 and early 2011 when the CervicalCheck programme was in its infancy. A lot has changed over the last 10 years regarding cervical cancer prevention in Ireland e.g. increased knowledge and awareness among women and the general population of cervical screening, HPV infection and the HPV vaccines. From the perspective of GPs and practice nurses, influences on their cervical-screening related clinical behaviours may also have changed. Using the TDF for analysis meant that we did not have to select, *a priori*, a single psychological theory of behaviour, thus minimising the likelihood of missing important influences on clinical behaviour. However, the TDF does not specify relationships between the domains ^38, 39^. Finally, while we were only able to determine in a qualitative way which of the domains were likely to be the most important drivers of clinical behaviour, the study was intended to be hypothesis **-**generating rather than hypothesis **-**testing.

## Conclusions

We identified - for the first time - primary care practitioners’ cervical-screening related clinical behaviours, their perceived roles and responsibilities, and the factors likely to influence behaviours. In addition to taking smears, practitioners considered it their responsibility to encourage women to have smears, but smeartaking was viewed as a predominantly female role. The results could inform development of strategies to: motivate and enable practitioners to encourage women to have smear/screening tests; and/or improve women’s access and experiences; these in turn, may help increase cervical screening uptake. However, engaging male practitioners with these initiatives may prove difficult.

## Data availability

### Underlying data

There are no quantitative data associated with this article. The audio files and transcripts generated during the current study are confidential. In the consent document, participants were not asked to consent to sharing of data beyond the research team and their collaborators. A comprehensive set of quotes reflecting the transcripts are available in Table 2. Researchers seeking to access the underlying data (i.e. audio files and transcripts) will need to apply directly to the Irish College of General Practitioners Research Ethics Committee for approval. The Committee can be contacted at research@icgp.ie. Should approval be granted, the authors are happy to facilitate access.

### Extended data

Figshare: Extended Data: Identifying ways to maximise cervical screening uptake: a qualitative study of GPs’ and practice nurses’ cervical cancer screening-related behaviours https://doi.org/10.6084/m9.figshare.14132105.v1 ^25^.

- Interview topic guide
- SRQR checklist

Data are available under the terms of the Creative Commons Attribution 4.0 International license (CC-BY 4.0).
