# Supplementary material for: Identifying ways to maximise cervical screening uptake: a qualitative study of GPs’ and practice nurses’ cervical cancer screening-related behaviours
Source: HRB Open Res. 2021 May 5;4:44. [Version 1] doi: 10.12688/hrbopenres.13246.1 (PMC8370130; doi:10.12688/hrbopenres.13246.1)
Supplement: Peer review history

# Peer review: Reviewer response for version 1

**DOI:** 10.21956/hrbopenres.14409.r29993

**Published:** 2021-08-16

**Authors:** Suhailah M Ali

**Affiliations:** 1 College of Nursing, University of Kirkuk, Kirkuk, Iraq

This paper presents a very important issue concerning primary care practitioners’ and practice nurses’ perceived roles and responsibilities, in terms of cervical-screening related clinical behaviours. The study aimed to determine factors likely to influence clinical behaviours. 19 GPs and 14 practice nurses in Ireland participated. Authors utilized a well-established Theoretical Domains Framework (TDF) to analyse the interview transcripts. The table describing the included 12 possible theoretical domains was useful to gain an in-depth understanding of the issues around cervical screening from practitioners’ perspectives. The findings of this study could inform development of interventions to enhance practitioners’ clinical behaviours to promote cervical screening uptake.

**Introduction:** The research question clearly identifies the importance of factors influencing primary care practitioners’ and practice nurses’ cervical screening-related clinical behaviours to encourage cervical screening uptake. The introduction is well written and provides clear justification.

**Methods: **The setting of the study is well described **.** It is evident to the reader what current practices are regarding cervical screening in Ireland with regards to switching to the primary HPV testing in line with recent international recommendations. Authors described well how the participants were recruited and how it evolved as the study progressed. Recruitment continued until the decision was reached that there was sufficient depth of information to meet the purpose of the study.

**Result**: The results of this qualitative study are well presented to address the research questions. Table 2 enabled us to get an in depth snapshot of the challenging contexts in which the practitioners’ and practice nurses’ worked.

 However, there are few minor points I noticed that ought to be rectified:

- In the result section, page 5: Table 1 shows 19 GPs and 14 practice nurses participated in the study. Whereas in the abstract page 1, result section: authors stated 14 GPs and 19 practice nurses participated? This need to be consistent.

**Discussion:** Authors clearly explained how this study has attempted to identify factors likely to influence practitioner’s clinical behaviours and they made explicit statements from their study to compare with the existing literature. The implications for practice seemed appropriate and key messages are accurate indicating that practitioners are willing to consider a more active role in encouraging women to uptake cervical smears. Limitations of the paper are well presented. However, I can not see suggestions for further investigation in the area of practice which leaves the reader with the implication of the phenomenon being researched and no onward plan for future research.

 In summary, this is a professional written paper with high level of in-depth analysis and a well-structured presentation of arguments. The findings make significant contribution to the field of cervical screening uptake.

Is the work clearly and accurately presented and does it cite the current literature?

Yes

If applicable, is the statistical analysis and its interpretation appropriate?

Not applicable

Are all the source data underlying the results available to ensure full reproducibility?

Yes

Is the study design appropriate and is the work technically sound?

Yes

Are the conclusions drawn adequately supported by the results?

Yes

Are sufficient details of methods and analysis provided to allow replication by others?

Yes

Reviewer Expertise:

cervical cancer screening , qualitative studies, mixed methods studies, systematic reviews

I confirm that I have read this submission and believe that I have an appropriate level of expertise to confirm that it is of an acceptable scientific standard.

## References

[1] : A guide to using the Theoretical Domains Framework of behaviour change to investigate implementation problems. *Implementation Science*.2017;12(1) : 10.1186/s13012-017-0605-910.1186/s13012-017-0605-928637486PMC5480145

# Peer review: Reviewer response for version 1

**DOI:** 10.21956/hrbopenres.14409.r29408

**Published:** 2021-06-01

**Authors:** John Campbell

**Affiliations:** 1 Collaboration for Academic Primary Care (APEx), University of Exeter, Exeter, UK

Thank you for the opportunity to review this interesting article setting out to address three clear aims in respect of the observed decline in cervical screening. A qualitative study of 14 GPs and 19 practice nurses is reported.

**Abstract** - The abstract is broadly acceptable although does lack some precision and clarity in respect of the presentation of results. Exactly what behaviours are being referred to line 3? In addition, a very substantial claim is made round the originality of this study. Although this is not my principal area of expertise or interest I’d be very surprised if this was the first such study to explore primary care practitioners’ behaviours in respect of cervical screening

**Introduction** – the introduction presents a compelling and well-articulated case for the paper, noting the decline in cervical screening uptake, some potential issues impacting on the likelihood that women will respond to screening requests and the previous experience on doctors (although I would want to be reassured that this was comprehensive).

**Methods** – the setting of the study is well described, including a short summary of the Irish health Service. The situation is described where the responsibility for taking smears has moved from GPs to practice nurses. Certainly for some GPs this is a frustration, and may be undermining of the whole cervical cytology programme.

 The authors clearly outline their approach to sampling for the qualitative research, but it seems likely that participants would be subject to selection bias having taken part in previous related research (for GPs). The approach to practice nurses seems more comprehensive, with recruitment via a conference or to nurse development coordinators.

 The interview procedures are standard, and well described. Recruitment continued until saturation was evident. Analysis involved a framework approach with theoretical domains framework as the coding framework.

**Results** - recruitment appears to have covered a range of participants as defined by gender, geographical distribution, practice setting, clinical experience etc.

 A range of quotes are provided in respect of roles and responsibilities in respect of smear taking.

 A useful table (2) identifies factors influencing behaviours relating to cervical screening, accompanied by useful quotes. The results focus on belief as well as organisation factors relating to time, clinical behaviours, and external social influences.

**Discussion** – the findings are neatly summarised and presented in the context of a reasonable comparison with relevant international literature. Key issues identified in the results are followed through in the discussion and a comprehensive contextualisation of the findings is presented and discussed. A range of implications for practice are presented although I was surprised that some of the anticipated views of male GPs is not reflected in the implications for practice – for many experienced GPs, taking smears was a normal part of routine practice and it is possibly to the detriment of overall clinical care, including to the experience of women, that this clinical procedure is now seen to be within the domain of nurses or female GPs.

 A range of strengths and limitations of the work are presented and reasonable conclusions are drawn (although, again, I would question whether this is the first such study in this area).

 Overall however, this is a carefully conducted piece of important research which is well presented, well written, and provides an interesting range of significant findings. I have no hesitation in supporting its publication with only minor changes.

Is the work clearly and accurately presented and does it cite the current literature?

Yes

If applicable, is the statistical analysis and its interpretation appropriate?

Not applicable

Are all the source data underlying the results available to ensure full reproducibility?

Yes

Is the study design appropriate and is the work technically sound?

Yes

Are the conclusions drawn adequately supported by the results?

Yes

Are sufficient details of methods and analysis provided to allow replication by others?

Yes

Reviewer Expertise:

Primary care; access; quality

I confirm that I have read this submission and believe that I have an appropriate level of expertise to confirm that it is of an acceptable scientific standard.
